# Complexity of coronary artery disease and the release of cardiac biomarkers after CABG

**DOI:** 10.3389/fcvm.2024.1345439

**Published:** 2024-02-01

**Authors:** Leo Pölzl, Ronja Lohmann, Philipp Sterzinger, Felix Nägele, Jakob Hirsch, Michael Graber, Clemens Engler, Jonas Eder, Hannes Abfalterer, Hanno Ulmer, Andrea Griesmacher, Michael Grimm, Nikolaos Bonaros, Elfriede Ruttmann-Ulmer, Johannes Holfeld, Can Gollmann-Tepeköylü

**Affiliations:** ^1^ Department of Cardiac Surgery, Medical University of Innsbruck, Innsbruck, Austria; ^2^ Department of Statistics, University of Warwick, Coventry, United Kingdom; ^3^ Department for Medical Statistics, Informatics and Health Economics, Medical University of Innsbruck, Innsbruck, Austria; ^4^ Central Institute of Clinical Chemistry and Laboratory Medicine, Medical University of Innsbruck, Innsbruck, Austria

**Keywords:** biomarker release, perioperative risk stratification, CABG, SYNTAX Score, cardiac surgery

## Abstract

**Objective:**

In patients with complex coronary artery disease (CAD) undergoing cardiac surgery, myocardial protection might be impaired due to microvascular obstruction, resulting in myocardial injury and subsequent biomarker release. Therefore, this study investigated the correlation between the complexity of CAD, reflected by the SYNTAX Score, and the release of cardiac biomarkers after CABG.

**Methods:**

In a consecutive series of 919 patients undergoing isolated CABG SYNTAX scores I and II were calculated to assess the complexity of CAD. Levels of high sensitivity cardiac troponin T (hs-cTnT) and creatine kinase-myocardial band (CK-MB) were routinely measured once before and serially after surgery. Patients were divided into tertiles according to their SYNTAX Scores I and II. Spearman correlations and regression models were performed to measure the degree of association between the release of hs-cTnT and CK-MB and the SYNTAX Scores.

**Results:**

Patients with a higher SYNTAX Score I had more comorbidities reflected in a higher EuroSCORE II. Preoperatively, higher levels of cardiac biomarkers were found in patients with higher SYNTAX Score II. No correlation was observed between hs-cTnT, CK-MB and SYNTAX Score I or II. Regression models did not show any association between cardiac biomarkers and the complexity of CAD.

**Conclusion:**

The complexity of CAD is not associated with the release of cardiac biomarkers after CABG. Factors influencing postoperative biomarker release need to be elucidated in future trials to include postoperative biomarker release into risk stratification models predicting outcome after cardiac surgery.

## GRAPHICAL ABSTRACT

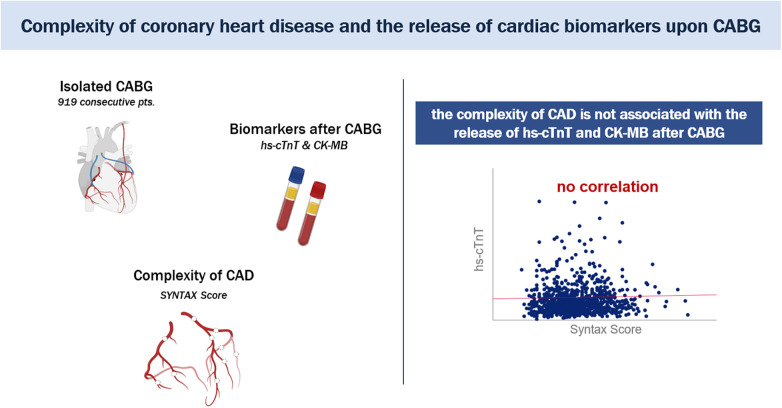

## Introduction

Coronary artery bypass grafting (CABG) is the most frequently performed cardiac surgery procedure worldwide. Almost half a million procedures are performed in the US and Europe annually (1, 2). Myocardial ischemia with cardioplegic arrest and subsequent reperfusion during cardiac surgery result in the release of cardiac biomarkers. Periprocedural myocardial infarction remains a serious complication resulting in severe impairment of patient prognosis (3). Released cardiac biomarker levels are crucial for the timely diagnosis of periprocedural myocardial infarction, enabling rapid action to reverse ischemia (3–5). Clinically relevant biomarker cut-offs after CABG surgery to diagnose periprocedural myocardial infarction are conversely debated. Besides their pivotal role in the diagnosis of perioperative myocardial infarction, postoperative biomarker levels as correlate for periprocedural myocardial injury are able to predict 30-day mortality after cardiac surgery (6).

Thoroughly performed cardioplegic arrest is crucial to protect the myocardium from ischemia during aortic cross clamping (7). Patients with complex coronary artery disease (CAD) often suffer from microvascular disease. Atherosclerotic lesions of the microvascular coronary bed might impair the efficacy of cardioplegia making the myocardium of these patients more susceptible for periprocedural myocardial injury.

The SYNTAX Score I is an angiographic tool grading the complexity of CAD (8, 9). Postoperative cardiac biomarker levels are influenced by the extent of heart failure, kidney and liver function (10–12). The SYNTAX Score II unites complexity of CAD with clinical characteristics, allowing to guide decision making between CABG and percutaneous coronary intervention (13). Both scores are associated with the outcome after CABG (14). High SYNTAX Score values are associated with increased cardiac biomarker levels upon percutaneous coronary intervention (15).

Factors influencing the release of cardiac biomarkers during cardiac surgery are still not completely understood. They include mechanical manipulation and cardiopulmonary bypass, cardioplegic arrest and ischaemia-reperfusion injury, ischaemia due to graft failure and even perioperative tachyarrhythmia (7, 16–21).

In patients with complex CAD undergoing cardiac surgery, myocardial protection might be impaired due to microvascular obstruction, resulting in myocardial injury and subsequent biomarker release.

Therefore, this study investigated the correlation between the complexity of CAD, reflected by the SYNTAX Score, and the release of cardiac biomarkers after CABG.

## Methods

### Study population and data collection

In a consecutive series of 928 patients undergoing isolated CABG at the Medical University of Innsbruck between 2012 and 2015 SYNTAX Score I and SYNTAX Score II were calculated. Patients with at least one measurement of postoperative hs-cTnT were analysed. No other patients were excluded, resulting in a cohort of 919 patients (Supplementary Figure S1). Indication for CABG surgery was discussed within the heart team, in accordance with current guidelines. The performing surgeons decided on the surgical strategy, including the choice and number of grafts used. General anaesthesia was induced with midazolam, esketamine, propofol, fentanyl, and rocuronium and continued with ihnhalation anaesthesia via sevoflurane (2%–2.5%, target MAC 0.8–1). CABG procedure was performed in in all patients on pump under cardioplegic arrest. Cardioplegic arrest was performed with mainly via antegrade cardioplegia, according to the surgeons' preference and if necessary retrograde administration was performed additionally of St. Thomas II cardioplegic solution was applied in a 20–25 min interval during surgery. Hs-cTnT was measured once preoperatively and serially within the first 72 h after surgery (4, 12, 24, 48, and 72 h) (hs-cTnT and CK-MB; mean number of postoperative measurements: 4.7). For statistical calculations the highest value measured was used. For hs-cTnT the Elecsys® Troponin T-high sensitive assay (Roche Diagnostics, Mannheim, Germany) and for CK-MB the Cobas® assay (Roche Diagnostics, Mannheim, Germany) was used. Survival data was obtained by the Austrian federal statistics institute (“*Statistik Austria*”*)* as well as patient health record database for non-Austrian patients.

This trial was performed in accordance with the Declaration of Helsinki and permission to use anonymized data without patient consent for this study was obtained from the Innsbruck Medical University Institutional Review Board (1233/2022).

### Statistical analysis

Categorical variables are presented as frequencies and proportions. Continuous variables are presented as medians and interquartile ranges. The cohort was divided into three tertiles according to SYNTAX Score I and SYNTAX Score II, respectively. Statistical analyses were performed for both scores separately. Kruskal–Wallis Test was used to analyse differences between the three tertiles. Levels of cardiac biomarkers upon surgery are presented in whisker blots with the 5–95 percentile. To assess the association between SYNTAX Scores and cardiac biomarker levels Spearman’s correlation and linear regressions were performed. In a sensitivity analyses elective cases were analysed only. *P* values < 0.05 were considered to indicate statistical significance. All statistical analyses were performed with SPSS Version 24 (IBM Corporation, Armonk, NY, USA) and GraphPad Prism version 9.0.

### Graphical abstract

The central image was created with *BioRender.com*.

## Results

### Study population

In total, 919 patients undergoing isolated CABG were included in the study. The median SYNTAX Score I was 28 [21–35] and the median SYNTAX Score II 30.2 [22.3–37.4], respectively. All patients were divided into three equally sized groups by SYNTAX Score I (<24 vs. 24–32 vs. >32) and SYNTAX Score II (<25.3 vs. 25.3–34.9 vs. >34.9) (Supplementary Table S1, Supplementary Figure S2).

In the upper SYNTAX Score I tertile more diabetic patients (23.7% vs. 29.8% vs. 34.0%, *p* = 0.019), with less history of smoking (50.0% vs. 42.1% vs. 39.9%, *p* = 0.030) were observed. LV-EF was lower (60% vs. 60% vs. 55%, *p* < 0.001), and patients were more symptomatic according to NYHA classification (*p* = 0.008) in patients with higher SYNTAX Scores (Table 1). History of myocardial infarction or PCI and their time point did not differ between groups (Table 1, Supplementary Table S3).

**Table 1:** Baseline characteristics of tertiles divided according to the SYNTAX score I.

	All patients *n* = 919	Lower tertile *n* = 304 (33.1%)	Middle tertile *n* = 309 (33.6%)	Upper tertile *n* = 306 (33.3%)	*p*-value
Demographic characteristics					
Sex (female)	154 (16.8%)	56 (18.4%)	55 (17.8%)	43 (14.1%)	0.294
Age (years)	68.31 [60.10–74.17]	67.31 [58.93–73.63]	68.38 [60.50–74.13]	69.39 [61.02–74.75]	0.107
BMI	27.0 [24.7–29.6]	26.8 [24.7–29.6]	27.0 [24.7–29.2]	27.2 [24.7–30.0]	0.510
Pre-existing conditions					
Diabetes	268 (29.2%)	72 (23.7%)	92 (29.8%)	104 (34.0%)	**0** **.** **019**
Dyslipidemia	877 (95.4%)	292 (96.1%)	294 (95.1%)	291 (95.1%)	0.817
History of smoking	404 (44.0%)	152 (50.0%)	130 (42.1%)	122 (39.9%)	**0**.**030**
Creatinin (mg/dl)	1.00 [0.86–1.16]	0.99 [0.86–1.13]	1.00 [0.85–1.20]	1.00 [0.86–1.17]	0.800
Current on dialysis	6 (1.3%)	2 (1.2%)	2 (1.3%)	2 (1.5%)	0.975
Stroke	72 (7.8%)	20 (6.6%)	28 (9.1%)	24 (7.8%)	0.520
Myocardial infarction	434 (47.2%)	136 (44.7%)	140 (45.3%)	158 (51.6%)	0.166
PCI	238 (25.9%)	83 (27.3%)	86 (27.8%)	69 (22.5%)	0.259
LV-EF (%)	60 [50–64]	60 [53–65]	60 [50–65]	55 [45–61]	**<0**.**001**
LV-EF grouped					**<0**.**001**
<20%	9 (1.0%)	4 (1.3%)	2 (0.6%)	3 (1.0%)	
21%–30%	33 (3.6%)	9 (3.0%)	8 (2.6%)	16 (5.2%)	
31%–50%	228 (24.8%)	57 (18.8%)	75 (24.3%)	96 (31.4%)	
>50%	649 (70.6%)	234 (77.0%)	224 (72.5%)	191 (62.4%)	
NYHA class					**0**.**008**
I	135 (14.7%)	46 (15.1%)	55 (17.8%)	34 (11.1%)	
II	368 (40.0%)	134 (44.1%)	120 (38.8%)	114 (37.3%)	
III	360 (39.2%)	108 (35.5%)	118 (38.2%)	134 (43.8%)	
IV	56 (6.1%)	16 (5.3%)	16 (5.2%)	24 (7.8%)	
Cardiac biomarkers					
Creatine kinase (U/L)	94.0 [66.5–135.0]	99.0 [70.0–136.5]	93.0 [67.5–134.0]	90.5 [63.8–131.0]	0.268
High sensitive troponin T (ng/L)	13.1 [8.8–27.9]	12.5 [7.9–22.8]	13.1 [9.1–29.5]	14.2 [9.6–30.4]	0.085

BMI, body mass index; COPD, chronic obstructive pulmonary disease; LV-EF, left ventricular ejection fraction; PCI, percutaneous coronary intervention.

The bold values indicate significant *p*-values < 0.05.

In the lower tertile of the SYNTAX Score II patients were more often female (19.4% vs. 18.4% vs. 12.5%, *p* = 0.046) and younger (57.3 vs. 69.6 vs. 74.9 years, *p* < 0.001). Patients in the upper tertile had a lower BMI (27.8 vs. 27.0 vs. 26.0, *p* < 0.001) and had less frequently a history of smoking (59.9% vs. 35.7% vs. 36.1%, *p* < 0.001). Patients with higher SYNTAX Score II showed higher creatinine levels (0.92 mg/dl vs. 0.97 mg/dl vs. 1.10 mg/dl, *p* < 0.001), had more often a history of previous strokes (3.6% vs. 7.9% vs. 12.1%, *p* < 0.001). Upper tertile patients had lower LV-EF (60% vs. 60% vs. 55%, *p* = 0.008) and were more symptomatic according to the NYHA classification (*p* < 0.001) (Table 2).

**Table 2:** Baseline characteristics of tertiles divided according to the SYNTAX score II.

	All patients *n* = 919	Lower tertile *n* = 309 (33.6%)	Middle tertile *n* = 305 (33.2%)	Upper tertile *n* = 305 (33.2%)	*p*-value
Demographic characteristics					
Sex (female)	154 (16.8%)	60 (19.4%)	56 (18.4%)	38 (12.5%)	**0**.**046**
Age (years)	68.31 [60.10–74.17]	57.34 [51.99–61.35]	69.63 [66.00–73.13]	74.86 [71.42–79.38]	**<0**.**001**
BMI	27.0 [24.7–29.6]	27.8 [25.3–30.4]	27.0 [25.0–29.9]	26.0 [24.0–28.7]	**<0**.**001**
Pre-existing conditions					
Diabetes	268 (29.2%)	77 (24.9%)	104 (34.1%)	87 (28.5%)	**0**.**042**
Dyslipidemia	877 (95.4%)	292 (94.5%)	295 (96.7%)	290 (95.1%)	0.394
History of smoking	404 (44.0%)	185 (59.9%)	109 (35.7%)	110 (36.1%)	**<0**.**001**
Creatinin (mg/dl)	1.00 [0.86–1.16]	0.92 [0.81–1.05]	0.97 [0.86–1.13]	1.10 [0.94–1.32]	**<0**.**001**
Current on dialysis	6 (1.3%)	1 (0.8%)	3 (1.9%)	2 (0.7%)	0.684
Stroke	72 (7.8%)	11 (3.6%)	24 (7.9%)	37 (12.1%)	**<0**.**001**
Myocardial infarction	434 (47.2%)	156 (50.5%)	137 (44.9%)	141 (46.2%)	0.352
PCI	238 (25.9%)	81 (26.2%)	77 (25.2%)	80 (26.2%)	0.951
LV-EF (%)	60 [50–64]	60 [51–65]	60 [50–65]	55 [45–62]	**0**.**008**
LV-EF grouped					**<0**.**001**
<20%	9 (1.0%)	4 (1.3%)	1 (0.3%)	4 (1.3%)	
21%–30%	33 (3.6%)	8 (2.6%)	9 (3.0%)	16 (5.2%)	
31%–50%	228 (24.8%)	63 (20.4%)	72 (23.6%)	93 (30.5%)	
>50%	649 (70.6%)	234 (75.7%)	223 (73.1%)	192 (63.0%)	
NYHA class					**<0**.**001**
I	135 (14.7%)	56 (18.1%)	44 (14.4%)	35 (11.5%)	
II	368 (40.0%)	140 (45.3%)	137 (44.9%)	91 (29.8%)	
III	360 (39.2%)	101 (32.7%)	109 (35.7%)	150 (49.2%)	
IV	56 (6.1%)	12 (3.9%)	15 (4.9%)	29 (9.5%)	
Cardiac biomarkers					
Creatine kinase (U/L)	94.0 [66.5–135.0]	98.0 [67.0–144.0]	94.0 [70.0–135.0]	90.0 [63.0–128.0]	0.169
High sensitive troponin T (ng/L)	13.1 [8.8–27.9]	10.3 [6.9–19.7]	12.1 [8.3–23.0]	17.5 [11.2–31.2]	**<0**.**001**

BMI, body mass index; COPD, chronic obstructive pulmonary disease; LV-EF, left ventricular ejection fraction; PCI, percutaneous coronary intervention.

Values are median [interquartile range] or *n* (%).

The bold values indicate significant *p*-values < 0.05.

### Preoperative cardiac biomarker levels and perioperative characteristics

Preoperatively, 13.1 [8.8–27.9] ng/L for hs-cTnT were measured. No differences were observed between the tertiles according to the SYNTAX Score I (12.5 vs. 13.1 vs. 14.2 ng/L, *p* = 0.085) (Table 1). Patients in the upper tertile according to SYNTAX Score II showed differences in their hs-cTnT levels (10.3 vs. 12.1 vs. 17.5 ng/L, *p* < 0.001) (Table 2).

EuroSCORE II was higher in upper tertile patients according to SYNTAX Score I (2.3 vs. 2.3 vs. 2.8, *p* = 0.001) and SYNTAX Score II (1.2 vs. 2.0 vs. 4.3, *p* < 0.001). Patients in the upper tertile of the SYNTAX Score I received more grafts (*p* < 0.001) and a longer cross clamp and perfusion times were observed (CCT: 66 vs. 66 vs. 70 min, *p* = 0.001, RPT: 110 vs. 110 vs. 121 min, *p* < 0.001) (Table 3). In patients with higher SYNTAX Score II no differences in the number of used grafts was found (*p* = 0.391). Cross clamp time was shorter in upper tertile patients (71 vs. 68 vs. 64 min, *p* = 0.003), but no differences were found in the perfusion time (116 vs. 115 vs. 112 min, *p* = 0.343) (Table 4).

**Table 3:** Perioperative characteristics of tertiles divided according to the SYNTAX score I.

	All patients *n* = 919	Lower tertile *n* = 304 (33.1%)	Middle tertile *n* = 309 (33.6%)	Upper tertile *n* = 306 (33.3%)	*p*-value
Surgical procedure					
Status					0.263
*Salvage*	1 (0.1%)	1 (0.3%)	0	0	
*Emergency*	21 (2.3%)	8 (2.7%)	4 (1.3%)	9 (3.0%)	
*Urgent*	203 (22.3%)	63 (21.0%)	65 (21.2%)	75 (24.8%)	
*Elective*	684 (75.2%)	228 (76.0%)	237 (77.5%)	219 (72.3%)	
EuroSCORE II	2.5 ± 3.7	2.3 ± 3.8	2.3 ± 2.9	2.8 ± 4.1	**0**.**001**
Cross clamp time (min)	67 [54–82]	66 [51–83]	66 [53–91]	70 [58–84]	**0**.**001**
Perfusion time (min)	114 [95–137]	110 [90–134]	110 [92–134]	121 [104–142]	**<0**.**001**
Number of grafts	3 [3–4]	3 [3–4]	3 [3–4]	4 [3–4]	**<0**.**001**
Postoperative course					
Creatine kinase (U/L)	531.0 [382.0–743.0]	527.0 [393.3–751.5]	519.0 [372.1–689.0]	558.0 [379.3–793.3]	0.208
CK-MB (U/L)	48.0 [36.0–62.0]	48.0 [36.0–63.0]	44.0 [34.0–58.0]	52.0 [40.0–67.3]	**<0**.**001**
High sensitive troponin T (ng/L)	1,048.5 [670.4–1,584.5]	1,070.5 [657.7–1,624.8]	964.6 [648.1–1,373.5]	1,112.5 [727.5–1,733.5]	**0**.**031**
Dialysis or hemofiltration	68 (7.4%)	22 (7.2%)	26 (8.4%)	20 (6.5%)	0.668
ECMO	18 (2.0%)	8 (2.6%)	4 (1.3%)	6 (2.0%)	0.490
Death within 30 days	17 (1.8%)	7 (2.3%)	4 (1.3%)	6 (2.0%)	0.641

Values are mean ± SD, median [interquartile range] or *n* (%).

The bold values indicate significant *p*-values < 0.05.

**Table 4:** Perioperative characteristics of tertiles divided according to the SYNTAX score II.

	All patients *n* = 919	Lower tertile *n* = 309 (33.6%)	Middle tertile *n* = 305 (33.2%)	Upper tertile *n* = 305 (33.2%)	*p*-value
Surgical procedure					
Status					0.126
*Salvage*	1 (0.1%)	0	0	1 (0.3%)	
*Emergency*	21 (2.3%)	4 (1.3%)	7 (2.3%)	10 (3.3%)	
*Urgent*	203 (22.3%)	65 (21.3%)	61 (20.2%)	77 (25.5%)	
*Elective*	684 (75.2%)	236 (77.4%)	234 (77.5%)	214 (70.9%)	
EuroSCORE II	2.5 ± 3.7	1.2 ± 1.0	2.0 ± 1.7	4.3 ± 5.6	**<0**.**001**
Cross clamp time (min)	67 [54–82]	71 [56–88]	68 [55–81]	64 [51–79]	**0**.**003**
Perfusion time (min)	114 [95–137]	116 [98–141]	115 [95–135]	112 [94–135]	0.343
Number of grafts	3 [3–4]	3 [3–4]	3 [3–4]	3 [3–4]	0.391
Postoperative course					
Creatine kinase (U/L)	531.0 [382.0–743.0]	595.0 [432.5–857.0]	500.0 [353.0–724.5]	485.0 [343.5–695.0]	**<0**.**001**
CK-MB (U/L)	48.0 [36.0–62.0]	48.0 [35.5–62.5]	47.0 [36.0–64.0]	49.0 [37.0–62.0]	0.649
High sensitive troponin T (ng/L)	1,048.5 [670.4–1,584.5]	1,022.5 [637.9–1,538.8]	1,015.0 [669.6–1,585.0]	1,094.0 [722.7–1,629.0]	0.285
Dialysis or hemofiltration	68 (7.4%)	9 (2.9%)	25 (8.2%)	34 (11.1%)	**<0**.**001**
ECMO	18 (2.0%)	1 (0.3%)	7 (2.3%)	10 (3.3%)	**0**.**027**
Death within 30 days	17 (1.8%)	2 (0.6%)	7 (2.3%)	8 (2.6%)	0.150

Values are mean ± SD, median [interquartile range] or *n* (%).

The bold values indicate significant *p*-values < 0.05.

### Release of cardiac biomarkers after cardiac surgery

**Figure 1:**
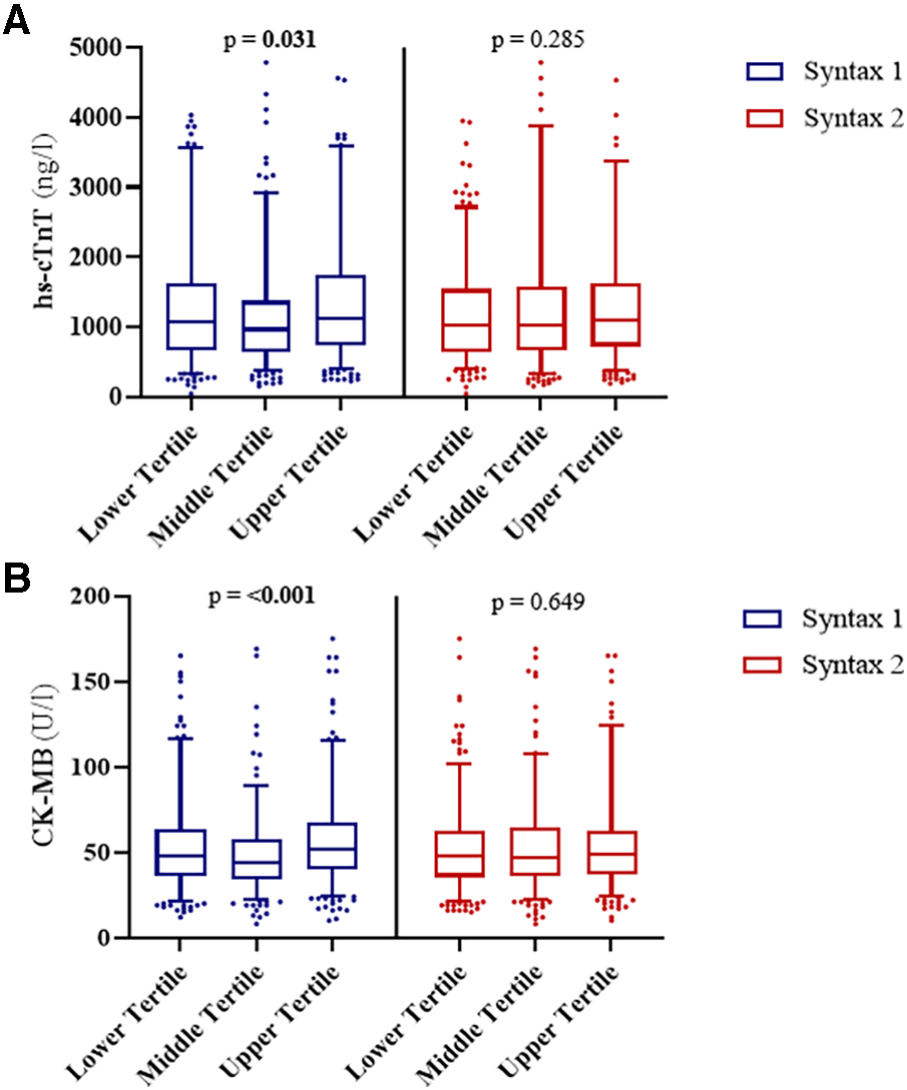
Release of cardiac biomarkers upon cardiac surgery. Patients were divided into three groups according to their SYNTAX score and SYNTAX score II results. No differences were observed in the levels of (**A**) hs-cTnT after procedure between SYNTAX score II groups (*p* = 0.285), but for SYNTAX score groups (*p* = 0.031). No differences were observed in the levels of (**B**) CK-MB between SYNTAX score II groups (*p* = 0.649), but for SYNTAX score groups (*p* < 0.001).

The overall postoperative peak hs-cTnT was 1,049 [670–1,585] ng/L and peak CK-MB was 48 [36–62] U/L (Table 3). hs-cTnT and CK-MB levels were highest in patients in the upper tertile according to SYNTAX Score I (hs-cTnT: 1,071 vs. 965 vs. 1,113 ng/L, *p* = 0.031; CK-MB: 48 vs. 44 vs. 52 U/L, *p* < 0.001) (Table 3, Figure 1**)**.

There was no difference in hs-cTnT (1,023 vs. 1,015 vs. 1,094 ng/L, *p* = 0.285) or CK-MB levels (48 vs. 47 vs. 49 U/L, *p* = 0.649) in the different SYNTAX Score II tertiles (Table 4, Figure 1). Patients in the upper tertile had lower CK levels (595 vs. 500 vs. 485 U/L, *p* < 0.001), an increased rate of dialysis or hemofiltration (2.9% vs. 8.2% vs. 11.1%, *p* < 0.001) and more need for ECMO support (0.3% vs. 2.3% vs. 3.3%, *p* = 0.027) (Table 4).

### Association between SYNTAX scores and post-operative cardiac biomarkers

**Figure 2:**
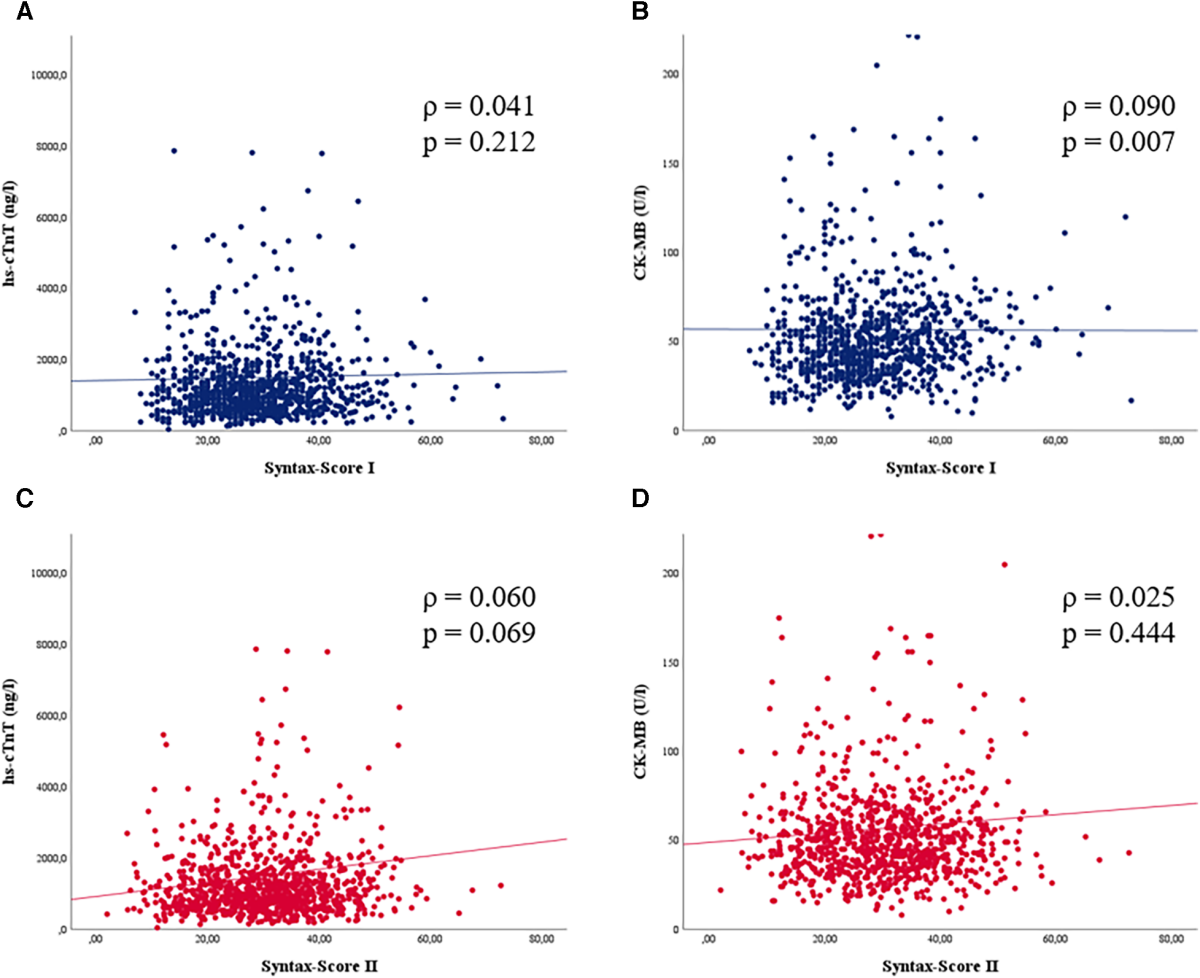
Scatter blots of postoperative hs-cTnT, CK-MB and the SYNTAX score I and II. Levels of (**A**) hs-cTnT and (**B**) CK-MB were correlated with SYNTAX score I (blue). No correlation was observed for both cardiac biomarkers. Neither were correlations observed for the SYNTAX score II (red) with (**C**) hs-cTnT and (**D**) CK-MB neither.

No correlation was observed between the SYNTAX Score I and post-operative hs-cTnT (*ρ* = 0.041) or CK-MB (*ρ* = 0.090) after CABG. Moreover, the cardiac biomarkers did not correlate with SYNTAX Score II (hs-cTnT: *ρ* = 0.060; CK-MB: *ρ* = 0.025) (Table 5 and Figure 2). There was no association for any pairing observed in a linear regression model (Table 5). In a sensitivity analysis, we repeated the investigation in elective cases only. Again, no association was observed between SYNTAX Scores and biomarkers (Supplementary Table S2).

**Table 5:** Correlations and associations between SYNTAX score I/SYNTAX score II and cardiac biomarkers upon cardiac surgery.

Correlations		
hs-cTnT	*ρ*	*p*-value
Syntax 1	0.041	0.212
Syntax 2	0.060	0.069
CK-MB		
Syntax 1	0.090	**0**.**007**
Syntax 2	0.025	0.444
Associations		
hs-cTnT	*R*²	*p*-value
Syntax 1	0.000	0.768
Syntax 2	0.004	0.053
CK-MB		
Syntax 1	0.002	0.959
Syntax 2	0.002	0.136

The bold values indicate significant *p*-values < 0.05.

## Discussion

In this study we investigated the influence of the complexity of coronary artery disease- defined by the SYNTAX Scores—on the release of cardiac biomarkers after CABG. The main finding of this study was that there was no correlation or association between the postoperative release of hs-cTnT or CK-MB and the SYNTAX Score or SYNTAX Score II.

However, we could observe decreased left ventricular function in patients in the upper tertiles of the SYNTAX Scores. In patients with a higher SYNTAX Score II increased levels of hs-cTnT were observed preoperatively.

The preoperative differences in patients’ characteristics with a higher SYNTAX Score were expected. Increased biomarker levels in these patients may result from the complexity and degree of the CAD or the decreased left ventricular function (10, 11). The SYNTAX Score II includes patients' characteristics (gender, age, COPD, LV-EF, CrCL) similar to the EuroSCORE II (22). Therefore, it was not surprising that patients with elevated SYNTAX score II were also found to have elevated EuroSCORE II scores. We believe that the increased rates of hemofilatation or dialysis, as well as the rate of ECMO support upon CABG, are due to patient comorbidities rather than to the complexity of CAD.

The SYNTAX Scores are a crucial tool for decision making between CABG and percutaneous coronary intervention and are associated with the outcome after CABG (13, 14). Levels of released cardiac troponin and CK-MB predict prognosis of CABG patients (6, 23). High SYNTAX Score values are associated with increased cardiac biomarker levels upon percutaneous coronary intervention (15).

Recent studies show that postoperative cardiac troponin levels as correlate for periprocedural myocardial injury are able to predict 30-day mortality after cardiac surgery (6, 24). Factors influencing the release of cardiac biomarkers during cardiac surgery, as cardiopulmonary bypass, cardioplegic arrest and ischaemia-reperfusion injury, are still not completely understood (17–19). Thoroughly performed cardioplegic arrest is crucial for the outcome after cardiac surgery. In off-pump patients, without cardioplegic arrest, remarkable lower values of cardiac biomarkers are observed postoperatively (7). We hypothesized that the distribution of the cardioplegia is more difficult in patients with complex CAD. The impaired myocardial protection would lead to and increased release of cardiac biomarkers as a sign of myocardial injury. Unexpectedly, we could not observe any association between the complexity of the CAD and the release of hs-cTnT or CK-MB. As no real trend could be observed in over 900 patients, we consider it unlikely that a higher number of cases would have much of an impact.

The SYNTAX Score II additionally reflects the patients' comorbidities. Interestingly, even in patients with a higher SYNTAX Score II, no differences were observed. Therefore, it seems unlikely that any comorbidities increase the release of cardiac biomarkers.

Other factors like completeness of revascularization might contribute to postoperative biomarker release and should be the subject of future research.

We could demonstrate in an earlier study, that multiple modalities for the diagnosis of clinical relevant pMI are more favorable than isolated biomarker release (3). Nevertheless, we also could demonstrate, that the release of cardiac biomarkers is associated with the 30-day mortality after cardiac surgery (24). We agree fully, that a release of biomarkers should be used as a trigger for further modalities to diagnose pMI. At which cut-off or at which release dynamic further modalities should be performed has to be investigated in further studies.

After CABG, hs-cTnT levels of approximately 1,000 ng/L are measured compared to 15 ng/L before surgery. We hypothesize that in statistical analyses this massive release potentially masks responsible factors, which probably strongly influence the release of cardiac biomarkers as mechanical manipulation, cardiopulmonary bypass and ischaemia-reperfusion injury during cardiac surgery are still not completely understood.

## Conclusion

Postoperative cardiac biomarker release is crucial to identify patients at risk after cardiac surgery. We could demonstrate that the complexity of coronary artery disease is not associated with the release of cardiac biomarkers. This finding is surprising as we hypothesized that patients with more complex coronary artery disease might be more susceptible for perioperative myocardial ischemia. Our findings implicate that patients with more complex coronary artery disease do not need higher perioperative cardiac biomarker cut-offs for the diagnosis of perioperative myocardial injury. In order to include postoperative biomarker release into risk stratification models, factors influencing biomarker release after surgery need to be elucidated in detail, as they remain largely unknown.

This includes factors such as sex, completeness of revascularization, myocardial mass and different cardioplegia strategies. If we understand exactly how myocardial injury occurs, we can also define the thresholds for when PMI occurs and when additional diagnostic testing modalities are needed.

### Limitations

This study has all the limitations of a single center observation. With a cohort size of over 900 patients, not even a trend of correlation could be observed. Therefore, we believe that an increase in cohort size or expansion to multiple centers would not result in a massively different outcome. This study only included patients undergoing on-pump surgery, as this represents the standard of care in our center. However, biomarker release after off-pump surgery differs markedly, therefore, the results of this study cannot be applied to patients undergoing off-pump surgery (7). Moreover, the used cardioplegia regime might also effect the biomarker release kinetics. In this manuscript, we have investigated the correlation of SYNTAX score with CK, CK-MB and hs-TnT release. There are other biomarkers for cardiac injury including platelet-related biomarkers, cystatin C, CRP, H-FABP and myoglobin (7). Due to the retrospective nature of our study, we only investigated the correlation of the SYNTAX Score with available biomarkers. Future studies are needed to investigate a possible correlation of the SYNTAX Score with other biomarkers not investigated in our study.

## Data Availability

The data analyzed in this study is subject to the following licenses/restrictions: the data underlying this article will be shared on reasonable request to the corresponding author. Requests to access these datasets should be directed to can.gt@i-med.ac.at.
