# Supplementary material for: Complexity of coronary artery disease and the release of cardiac biomarkers after CABG
Source: Front Cardiovasc Med. 2024 Feb 1;11:1345439. doi: 10.3389/fcvm.2024.1345439 (PMC10870426; doi:10.3389/fcvm.2024.1345439)

Supplemental material to

"Complexity of coronary artery disease and the release of cardiac biomarkers after CABG"

Supplemental Table 1: Grouping of cohort

	Syntax 1		Syntax 2
Lower Tertile	<24.0	Lower Tertile	<25.3
Middle Tertile	24.0-32.0	Middle Tertile	25.3-34.9
Upper Tertile	>32.0	Upper Tertile	>34.9

Patients were divided into three groups according to their SYNTAX Score and SYNTAX Score II results.

Supplemental Table 2: Associations between SYNTAX Score / SYNTAX Score II and cardiac biomarkers upon cardiac surgery for elective cases only

**hs-cTnT**	R^2	p-val
Syntax 1	0.002	0.257
Syntax 2	0.003	0.137
**CK-MB**		
Syntax 1	0.003	0.143
Syntax 2	0.002	0.287

Supplemental Table 3: Occurrence of myocardial infarction and PCI prior to the surgery

SYNTAX Score

	All patients n = 919	Lower Tertile n= 304 (33.1%)	Middle Tertile n= 309 (33.6%)	Upper Tertile n= 306 (33.3%)	p-value
Myocardial infarction	434 (47.2%)	136 (44.7%)	140 (45.3%)	158 (51.6%)	0.166
MI time					0.376
<6h	10 (2.3%)	6 (4.4%)	1 (0.7%)	3 (1.9%)	
6-24h	10 (2.3%)	1 (0.7%)	4 (2.9%)	5 (3.2%)	
1-7d	55 (12.7%)	19 (14.0%)	13 (9.3%)	23 (14.6%)	
8-21d	73 (16.8%)	16 (11.8%)	23 (16.4%)	34 (21.5%)	
22-89d	126 (29.0%)	39 (28.7%)	50 (35.7%)	37 (23.4%)	
>90d	160 (36.9%)	55 (40.4%)	49 (35.0%)	56 (35.4%)	
PCI	238 (25.9%)	83 (27.3%)	86 (27.8%)	69 (22.5%)	0.259
PCI time					0.91
<6h	19 (8.0%)	7 (8.4%)	6 (7.0%)	6 (8.7%)	
>6h	219 (92.0%)	76 (91.6%)	80 (93.0%)	63 (91.3%)	

SYNTAX Score II

	All patients n = 919	Lower Tertile n= 309 (33.6%)	Middle Tertile n= 305 (33.2%)	Upper Tertile n= 305 (33.2%)	p-value
Myocardial infarction	434 (47.2%)	156 (50.5%)	137 (44.9%)	141 (46.2%)	0.352
MI time					0.138
<6h	10 (2.3%)	1 (0.6%)	2 (1.5%)	7 (5.0%)	
6-24h	10 (2.3%)	2 (1.3%)	5 (3.6%)	3 (2.1%)	
1-7d	55 (12.7%)	16 (10.3%)	18 (13.1%)	21 (14.9%)	
8-21d	73 (16.8%)	28 (17.9%)	17 (12.4%)	28 (19.9%)	
22-89d	126 (29.0%)	48 (30.8%)	44 (32.1%)	34 (24.1%)	
>90d	160 (36.9%)	61 (39.1%)	51 (37.2%)	48 (34.0%)	
PCI	238 (25.9%)	81 (26.2%)	77 (25.2%)	80 (26.2%)	0.951
PCI time					0.722
<6h	10 (2.3%)	8 (9.9%)	5 (6.5%)	6 (7.5%)	
>6h	10 (2.3%)	73 (90.1%)	72 (93.5%)	74 (92.5%)	

PCI = percutaneous coronary intervention.

Supplemental Figure 1: Flow chart

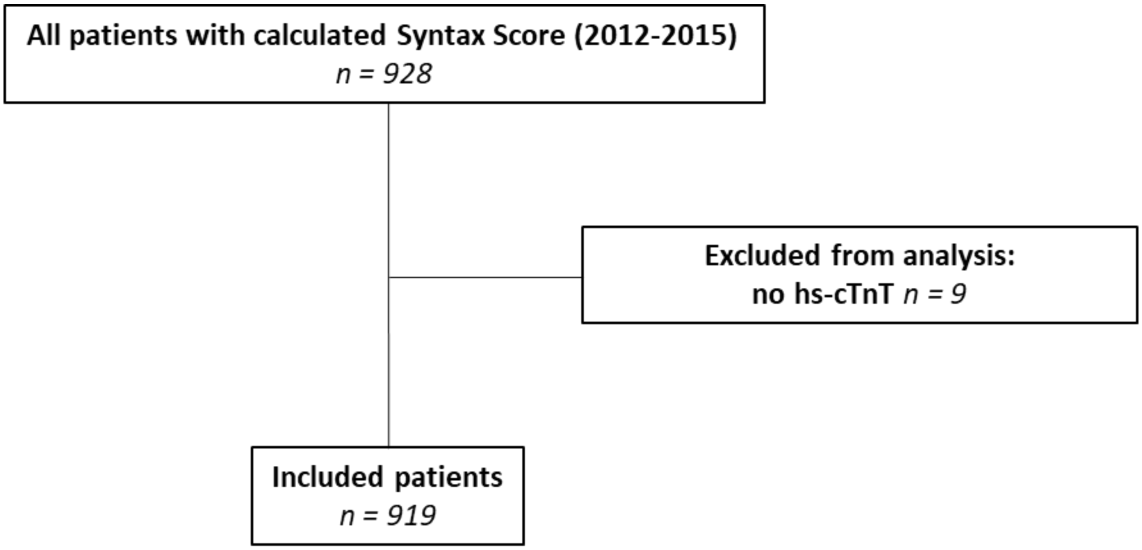

Supplemental Figure 2:

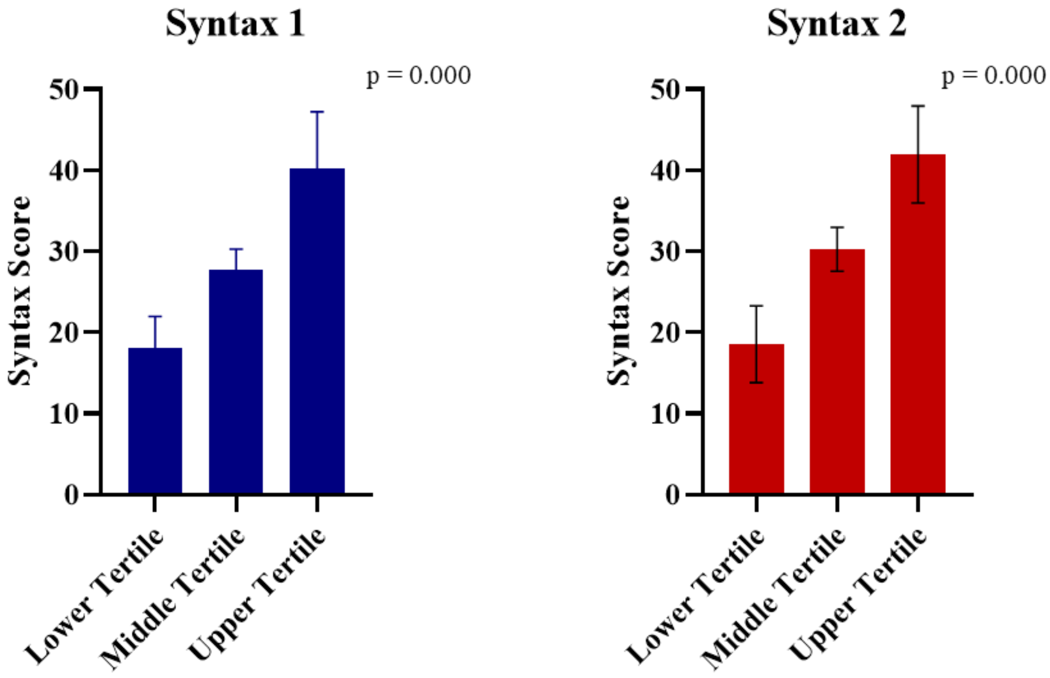

Supplement: Supplementary file 1 [file Datasheet1.pdf]
